# Translation, cross-cultural adaptation and validation of the Chinese version of supportive and palliative care indicators tool (SPICT-CH) to identify cancer patients with palliative care needs

**DOI:** 10.1186/s12904-024-01641-x

**Published:** 2025-01-07

**Authors:** Zhishan Xie, Siyuan Tang, Claire E. Johnson, Lin Xiao, Jinfeng Ding, Chongmei Huang

**Affiliations:** 1 Xiangya School of Nursing, Central South University, Changsha, China; 2 Palliative Aged Care Outcomes Program, Science, Medicine and Health, University of Wollongong, Wollongong, NSW Australia; 3 Nursing School, Southern Medical University, Guangzhou, China; 4 School of Nursing, Ningxia Medical University, 1160 Shengli Street of Xingqing District, Yinchuan, 750101 China

**Keywords:** Palliative care, SPICT, Chinese, Screening instrument

## Abstract

**Introduction:**

People diagnosed with cancer are the most frequent users of palliative care. However, there are no specific standards for early identifying patients with palliative care needs in mainland China. The Supportive and Palliative Care Indicators tool (SPICT) can identify patients with cancer who are in need of palliative care across healthcare settings.

**Objective:**

To translate, cross-cultural adapt the SPICT and validate it among cancer patients with palliative care needs in a Chinese healthcare context.

**Method:**

We translated and culturally adapted the SPICT from English into Chinese, following both Beaton’s and WHO’s recommendations: (1) initial translation, (2) synthesis, (3) back translation, (4) expert committee review, and (5) pretest. The psychometric properties (e.g., content validity, internal consistency, and inter-rater reliability) were analyzed. Convenience sample was used to recruit 212 hospitalized cancer patients between January and August 2023. Their needs were assessed by two nurses within 24hours to determine the inter-rater reliability and stability of the Mandarin version of SPICT (SPICT-CH).

**Results:**

All of 36 items were retained in response to expert review. The Scale-Content Validity Index/Ave (S-CVI/Ave) of the SPICT-CH was 0.98, demonstrating very strong content validity. The SPICT-CH exhibited good coherence (Cronbach’s alpha = 0.76) and reliability (Kappa = 0.71, 95% CI 0.71-0.72, *P* < 0.05).

**Conclusion:**

The SPICT-CH has good content validity and acceptable reliability among cancer patients within a Chinese hospital setting. This instrument can be effectively integrated into routine clinical practice to early identify patients who need palliative care in mainland China.

**Supplementary Information:**

The online version contains supplementary material available at 10.1186/s12904-024-01641-x.

## Introduction

China had the highest number of new cancer cases (4.57 million) and deaths (3 million) in the world in 2020, representing 23.7% and 30% of the global population, respectively [1, 2]. This has considerably increased the cancer burden in the country. Chinese patients with advanced cancer often experience increasing physical and psychological symptom severity and distress (e.g., pain and fear) during their final months of life [3–5]. Additionally, those patients have diverse needs and preferences regarding healthcare services  (e.g.,physical, psychological, spiritual, and social support) , towards the end of their lives. The disease trajectory and varied care needs imply the importance of introducing palliative care early in the treatment of cancer patients.

Palliative care is aimed at ensuring that patients facing life-threatening illnesses have access to tailored symptom management and appropriate holistic care to meet their needs and preferences [6]. In the early stages of disease progression, palliative care can be integrated with curative treatments to address the patient’s physical, psychological, social, and spiritual needs [7]. Early integration of palliative care and curative treatments could alleviate psychological distress (e.g., depression and anxiety), enhance quality of life, reduce symptoms, and decrease healthcare costs for advanced cancer patients [8–10]. The initial step in providing timely support is to identify individuals who are likely to benefit from early palliative care, and this holds significant importance in meeting the needs of patients requiring palliative care.

Our recent review identified several instruments that are used to ascertain patients who may benefit from palliative care [11]. Our findings revealed that Supportive and Palliative Care Indicators Tool (SPICT) had better clinical performance than other screening instruments [Gold Standards Framework Prognostic Indicator Guidance (GSF-PIG), the Necesidades Paliativas [PalliativeNeeds] (NECPAL), the RADboud indicators for Palliative Care needs (RADPAC), the Taiwanese version Palliative Care Screening Tool, Rainone and AnticiPal]. The SPICT was developed by a team of researchers at the University of Edinburgh in collaboration with clinicians from various healthcare settings, including hospitals, clinics and community settings [12]. The SPICT is a simple and easy-to-understand one-page instrument which helps clinicians to quickly and early identify individuals with palliative care needs [13].

The SPICT has 36 items and consists of three sections: (1) seven general indicators of deterioration in health, such as unplanned emergency admissions to hospitals, (2) seven sets of disease-specific clinical indicators (e.g., cancer, cardiovascular disease), and (3) seven healthcare recommendations which guide the clinicians to discuss the future care plan with the patient and their families based on the six-step guide in the REDMAP framework. TheSPICT has discernible advantages over alternative screening instruments. First, it does not include the Surprise Question, which to some extent reduces the possibility that the clinicians may omit patients who could benefit from early palliative care [14]. This is because the Surprise Question relies on clinicians’ intuition and experience to assess a patient’s prognosis before making a decision on palliative care provision. Second, it also presumes a suitable timeframe for patients to access palliative care. Third, it includes recommendations for palliative care plans, prompting the clinicians to address the patient’s needs and preferences in a structured manner. It is widely applicable across various healthcare settings, boasts extensive global use, hasbeen translated into 15 languages and has demonstrated with good reliability and validity in these linguistic settings. However, a Mandarinversion is not currently available.

Therefore, this study aims to (1) translate and cross-culturally adapt the Mandarinversion of SPICT (SPICT-CH) for use in mainland China, and (2) validate SPICT-CH among cancer patients in need of palliative care.

## Methods

This study is a component of a large project which is aimed tosupport early palliative care for Chinese cancer patients. We initially validated the instrument among cancer patients because theyare the largest population in need of palliative care in China.

The study involved two phases: the translation and cross-cultural adaptation of the SPICT and the assessment of the psychological properties of the SPICT-CH. In the first phase, all items were translated and cross-culturally adapted into Chinese. The second phase was conducted among cancer patients to validate the SPICT-CH. We, therefore, asked nurses to complete the generic indicators and cancer-specific items to identify cancer patients with unmet palliative care needs. All items were answered with “yes” or “no.” Patients were considered as needingpalliative care if they had two or more generic indicators of declining health along with one or more clinical indicators related to cancer.

### Translation progress and cross-cultural adaption

The Chinese translation and adaption of the SPICT followed the Beaton method and the WHO’s recommendation, which included the following five steps [15, 16].

#### Step 1: Initial translation

The instrument was independently translated the original version into Chinese by two researchers whose mother tongue is Chinese (X.Z. and T.M.) , resulting in documents I and II. They are native Chinese speakers with Master’s degrees in nursing. The translation followed Guillemin’s principle which emphasizes the preservation of cross-cultural equivalence between the original version and the target language version, and conformed to Chinese linguistic norms and expressions [17].

#### Step 2: Synthesis of the translation

Two researchers (X.Z. and T.M.) and a translation coordinator (D.J.) convened a meeting to compare documents I and II, analyzed the variations in expressions in relation to the original instrument, and ultimately forming document III.

#### Step 3: Back-translation

To verify the content of the instrument with the original, two additional translators (X.J. and John) independently conducted back-translations of document III. Subsequently, the research team compared the translated documents with the original instrument, identified the differences, and made necessary modifications to ensure that the Chinese translation is more than 90% consistent with the original. This resulted in version IV of the SPICT-CH. X.J. has a Ph.D. degree in nursing from Hong Kong, and John is a professional English translator.

#### Step 4: Expert committee review

A panel of 18 specialists in palliative care conducted two-rounds review of document IV to resolve discrepancies pertaining to semantic, conceptual, idiomatic, and empirical equivalence. Each specialist was asked to complete an electronic questionnaire and provide feedback on the document IV. The questionnaire includes three parts: the importance of early identification of palliative care, the introduction of the Mandarin version of SPICT, the purpose of the research and the content of the review. The revised document IV was sent back to the expert committee for further review. The expert committee consisted of six palliative care researchers, four palliative care physicians, one oncology physician, three oncology nurses, and four palliative care nurses.

#### Step 5: Pretest

To assess the understanding of each item and the feasibility of the instruments, seven nurses independently evaluated three adult cancer patients, by using the revised SPICT-CH. Those nurses had more than five years of clinical experience in the field of oncology, and they were from four different oncology units. After completing the evaluation, they were asked to rate the feasibility on a Likert-type scale, which was calibrated from one (extremely unsuitable) to ten (extremely feasible). The level of understanding was from one (very difficult) to 10 (very easy). We then conducted brief interviews with them to determine the presence of any ambiguities or uncertainties about the use of the SPICT-CH. Modifications were made based on the completion of the instrument, onsite interviews and feedback, leading to the final version of the SPICT-CH.

### Validation of the psychometric properties

First, an expert panel of five specialists was grouped to evaluate the content validity of the SPICT-CH. Experts rated each indicator on a four-point Likert scale (1 = not relevant; 2 = little relevant; 3 = relevant; 4 = highly relevant) with clarity, completeness, appropriateness, and relevance, and provided advice when necessary [18, 19]. The panel consisted of three oncology doctors and two palliative care specialists (one academic and one nurse). All of them had extensive experience in palliative care.

Subsequently, to assess the reliability of the SPICT-CH, we used PASS to calculate the sample size according to the guidelines proposed by Mohamad [20]. Selection of the Kappa coefficient was used to assess the level of inter-assessor agreement. We hypothesized that the Kappa coefficient of 0.60 would be similar to the previous study [21]. In order to achieve a sample test power of 80% with a 95% confidence interval of 0.05, 144 patients were needed. We recruited additional 16 patients with consideration of a 10% attrition rate. Therefore, a minimum sample size of 160 paired assessments was required. Finally, this study involved a convenience sample of 212 adult patients diagnosed with cancer from the oncology units. The patient’s nurse conducted assessments using the SPICT-CH. Assessments were repeated by another nurse within 24 h.

### Statistical analysis

Socio-demographic data of the patients were analyzed using descriptive statistics. The following analytical methods were used to test the validity and reliability of SPICT-CH. The SPSS 26.0 software was used for all statistical analyses.

### Content validity

We used Item-level CVI (I-CVI) and Scale-level CVI (S-CVI) to evaluate the content validity. Each Item-level CVI was adjusted for chance agreement using the multi-rater kappa statistic, following the criteria: fair = 0.40 to 0.59, good = 0.60 to 0.74, excellent > 0.74 [22]. The overall SPICT scale-level CVI (S-CVI) was calculated by using the average S-CVI (S-CVI Ave) and universal agreement Scale-level CVI (S-CVI/UA) recommended by Lynn [22]. The S-CVI/UA was calculated as a percentage of the total number of items rated by the expert panel as relevant/highly relevant (score of 3 or 4). The S-CVI Ave was the average of all items’ I-CVI. I-CVI > 0.78, S-CVI/UA > 0.80 and S-CVI Ave > 0.80 are the criteria for good content validity [23].

### Reliability

The Cronbach’s alpha was used to evaluate the internal consistency of the instrument, with a value exceeding 0.70 being recognized as indicative of acceptable reliability [24]. The muti-rater Fleiss-Kappa statistic (Kappa) was employed to determine the significance of the degree of inter-rater agreement. The Fleiss-kappa statistic is the ratio of the number of times that the nurses agreed on the SPICT-CH to the number of times that the nurses could agree (correction of chance agreement). The Fleiss-Kappa coefficient between 0.60 and 0.80 is considered good and moderate when between 0.40 and 0.60 [25].

### Feasibility

Feasibility was gauged by the time taken to complete the instrument and its acceptance among nurses, with completion time reported as an average. The perception of the instrument’s use in clinical practice and the understanding of the instrument’s content were obtained through a brief interview with the nurses in the pre-test process. All interviews are recorded with the consent of the interviewees. After the interview, the data were transcribed word for word and translated into English within 24 hours. The research team confirmed the accuracy of the translation in the form of group discussion and summarized into two themes: availability of the instrument and understanding of the content.

### Ethical considerations

This study was approved by the Nursing and Behavioral Medicine Research Ethics Review Committee of the Xiangya School of Nursing, Central South University in December 2022 (E2022209). We obtained permission from the developer of the original SPCIT for translation and cultural adaptation of the English into Chinese version.

## Results

### The translation and cultural adaptation

All of 36 items were retained after adaptation. The format was adjusted as follows: initial items linked by the terms “or” and “;” were divided into two or more subentries. For example, the indicators of deterioration in health, originally written as “Progressive weight loss; remains underweight; low muscle mass” is present as three sub-entries in SPICT-CH. If any one of the subentries was considered positive, the item was deemed positive. The Mandarin version of SPICT and the introduction of usage are attached in Appendix S1. The expert committee identified several concepts that required modification to address the Chinese context. The following describes the points of consensus reached by the expert review committee.

### Semantic equivalence

- The translation of the item “depends on others for care due to increasing physical and/or mental health problems” was not carried out literally. The panel believed that “physical and mental health problem” and “physical or mental health problem” emphasize, respectively, “symptoms complexity” and “physical functioning and emotional function”, which are two different dimensions. In addition, the experts reached a consensus to add “spiritual concerns” to the item because the presence or absence of spiritual concerns is an important element of palliative care. Consequently, to enhance clarity, the item was divided into two subentries: “Dependent on others for care due to increasing physical problems” and “Need for psychological or spiritual support due to increasing mental health or psychiatric problems, ” .

### Idiomatic equivalence

- The panel suggested adapting the term “limited reversibility” (可逆性有限) into “low probability of reversal” (逆转的可能性低).
- The panel’s recommendations led to adaptation of “life-limiting conditions“(限制生命的情况) into “terminal“(终末期).
- The panel adapted the term “specialist assessment“(专家评估) to " multidisciplinary team assessment” (多学科团队评估).

### Empirical equivalence

- The panel agreed to add the information of “oxygen partial pressure below 90mmhg” to the item “persistent hypoxia” to clarify the criteria for hypoxia.
- The experts concurred on the addition of “advance care planning” to the “future care plan” item to provide clarity regarding the form of future plans.

### Conceptual equivalence

- The panel reached a consensus that the term “optimal treatment” in the Chinese context did not make clear what treatment regimen is optimal. It was therefore redefined as “the intervention that produces the greatest value or maximizes utility for the patient”. This clarification has been incorporated as a supplementary explanation of the item.
- The panel agreed that the literal translation of “liver transplant is not possible” posed a difficulty in comprehension in Chinese. Therefore, it was translated as “unable or unsuitable for a liver transplant” and “or liver transplant failed” to enhance clarity and understanding within the Chinese context.

Subsequently, a pretest was conducted with seven nurses in the oncology units, resulting in a total of 25 completed assessments. The average time of completion was three minutes and 40 seconds. The average score for feasibility was 7.6 out of 10, and the average score for level of understanding was 8.3 out of 10. Two themes were identified from brief interview data: feasibility of application and easy-to-understand contents.

(1) Feasibility of application.

Oncology nurses perceived that integrating the SPICT-CH into their daily clinical practice was feasible and acceptable. Comments from nurses highlighted the instrument’s brevity, stating that it did not add to their workload. They acknowledged its utility in identifying patients with palliative care needs, even for those nurses who are already attentive to patient care.

*“The SPICT-CH is short and does not add to my workload. " (Nurse 1)*.

*“This instrument could help me identify patients with palliative care needs in my daily work. " (Nurse 3)*.

*“Although I usually pay close attention to patients*’ *care needs*,* using this instrument made it easy for me to identify their needs for palliative care. “(Nurse 6)*.

(2) Easy-to-understand contents.

All nurses agreed that the content of SPICT-CH was clear and easily comprehensible. While most items were well-understood, there were specific challenges related to subjective/comparative terms such as the judgment of ‘remains underweight or low muscle mass.’ Nurses with prior exposure to palliative care found the instrument more user-friendly than nurses with minimal palliative care experience.

*“…Except for ‘remains underweight or low muscle mass*,*’ which is a little hard to judge*,* the rest of the content is pretty well understood. “(Nurse 5 and 7)*.

*“Nurse who have attended or studied palliative care are more proficient in using SPICT-CH compared to those who have not been exposed to palliative care. " (Nurses 3*,*4 and 5)*.

*“I think that clarity regarding the concept of palliative care is essential*,* as a lack of understanding may pose difficulties in using SPICT-CH. " (Nurse 2)*.

Following discussions within the research team, no modifications were made to the contents of the instrument.

### The psychometric properties of SPICT-CH

A total of 424 matched assessments were completed for 212 cancer patients from oncology units of three hospitals in Hunan Province. One hospital specializes in cancer treatment, while the other two are general hospitals. All assessments took place in inpatient units. As shown in Table 1, ages of patients ranged from 20 to 81 years (mean = 56 years, SD = 11.09). The proportion of men and women was roughly equal. The three common types of cancers were lung cancer (32.5%), colorectal cancer (22.6%), and breast and gynecological malignancies (20.8%).

**Table 1:** Characteristics of patients

Variable	*N* (%)
Total patients	212(100%)
Gender Male Female	117(55.2%) 95(44.8%)
Age (years) ≤ 44 45–59 ≥ 60	32(15.1%) 96(45.3%) 84(39.6%)
Educational level Primary Junior Secondary Secondary Higher	76(35.8%) 98(46.2%) 23(10.9%) 15(7.1%)
Cancer type Lung Colorectal Breast and gynecological Nasopharyngeal Esophageal Gastric Liver Mixed Others	69(32.5%) 48(22.6%) 44(20.8%) 14(6.6%) 6(2.8%) 6(2.8%) 5(2.4%) 7(3.3%) 16(7.5%)
Pathological classification I II III IV unknown	4(1.9%) 45(21.2%) 77(36.3%) 82(38.7%) 4(1.9%)
Co-morbidities Yes No unknown	116(54.7%) 93(43.9%) 3(1.4%)
Treatment Operation Chemoradiotherapy	38(17.9%) 174(82.1%)
Re-admission within 30 days Yes No	77(36.3%) 135(63.7%)

### Content validity

Five palliative care experts participated in assessment of the content validity. Demographic characteristics of the experts are present in Appendix S2. As shown in Table 2, the multi-rater kappa statistics after adjusting for opportunity agreement were > 0.78 for all items, indicating excellent agreement among assessors. The S-CVI/ Ave and S-CVI/UA were 0.98 and 0.92, which suggested high relevance and good comprehensiveness of the items. The experts did not recommend removal of any items, indicating good acceptability of the items.

**Table 2:** Content validity of SPICT-CH on evaluation of the expert panel

Item	Expert rating					Number of experts with a rating of 3 or 4	I-CVI	Pc	Kappa-adjusted I-CVI	Evaluation
	A	B	C	D	E					
1–1 Unplanned hospital admission(s).	4	4	4	3	4	5	1.00	0.041	1.00	Excellent
1–2 Performance status is poor or deteriorating, with limited reversibility. (e.g., The person stays in bed or in a chair for more than half the day.)	4	4	3	3	4	5	1.00	0.041	1.00	Excellent
1–3 Depends on others for care due to increasing physical and/or mental health problems.	4	4	3	3	4	5	1.00	0.041	1.00	Excellent
1–4 The person’s carer needs more help and support.	4	4	3	4	4	5	1.00	0.041	1.00	Excellent
1–5 Progressive weight loss; remains underweight; low muscle mass.	4	4	4	4	4	5	1.00	0.041	1.00	Excellent
1–6 Persistent symptoms despite optimal treatment of underlying condition(s).	4	4	3	3	4	5	1.00	0.041	1.00	Excellent
1–7 The person (or family) asks for palliative care; chooses to reduce, stop or not have treatment; or wishes to focus on quality of life.	4	4	4	4	4	5	1.00	0.041	1.00	Excellent
2 − 1 Functional ability deteriorating due to progressive cancer.	4	4	3	4	4	5	1.00	0.041	1.00	Excellent
2–2 Too frail for cancer treatment or treatment is for symptom control.	4	4	3	4	4	5	1.00	0.041	1.00	Excellent
2–3 Unable to dress, walk or eat without help.	4	4	3	2	4	4	0.80	0.156	0.76	Excellent
2–4 Eating and drinking less; difficulty with swallowing.	4	4	3	3	4	5	1.00	0.041	1.00	Excellent
2–5 Urinary and faecal incontinence.	4	4	3	3	4	5	1.00	0.041	1.00	Excellent
2–6 Not able to communicate by speaking; little social interaction.	4	4	3	3	4	5	1.00	0.041	1.00	Excellent
2–7 Frequent falls; fractured femur.	4	4	3	4	4	5	1.00	0.041	1.00	Excellent
2–8 Recurrent febrile episodes or infections; aspiration pneumonia.	4	4	3	4	4	5	1.00	0.041	1.00	Excellent
2–9 Progressive deterioration in physical and/or cognitive function despite optimal therapy.	4	4	3	4	4	5	1.00	0.041	1.00	Excellent
2–10 Speech problems with increasing difficulty communicating and/or progressive difficulty with swallowing.	4	4	3	4	4	5	1.00	0.041	1.00	Excellent
2–11 Recurrent aspiration pneumonia; breathless of respiratory failure.	4	4	3	4	4	5	1.00	0.041	1.00	Excellent
2–12 Persistent paralysis after stroke with significant loss of function and ongoing disability.	4	4	3	2	4	4	0.80	0.156	0.76	Excellent
2–13 Heart failure or extensive, untreatable coronary artery disease; with breathless or chest pain at rest or on minimal effort.	4	4	3	4	4	5	1.00	0.041	1.00	Excellent
2–14 Severe, inoperable peripheral vascular disease.	4	4	3	2	4	4	0.80	0.041	1.00	Excellent
2–15 Severe, chronic lung disease; with breathlessness at rest or on minimal effort between exacerbations.	4	4	3	3	4	5	1.00	0.041	1.00	Excellent
2–16 Persistent hypoxia needing long term oxygen therapy.	4	4	3	4	4	5	1.00	0.041	1.00	Excellent
2–17 Has needed ventilation for respiratory failure or ventilation is contraindicated.	4	4	3	4	4	5	1.00	0.041	1.00	Excellent
2–18 Stage 4 or 5 chronic kidney disease (eGFR < 30 ml/min) with deteriorating health.	4	4	3	3	4	5	1.00	0.041	1.00	Excellent
2–19 Kidney failure complicating other life limiting conditions or treatments.	4	4	3	4	4	5	1.00	0.041	1.00	Excellent
2–20 Stopping or not starting dialysis.	4	4	3	4	4	5	1.00	0.041	1.00	Excellent
2-21 Cirrhosis with one or more complications in the past year: diuretic resistant ascites, hepatic encephalopathy, hepatorenal syndrome, bacterial peritonitis, recurrent variceal bleeds	4	4	3	4	4	5	1.00	0.041	1.00	Excellent
2–22 Liver transplant is not possible.	4	4	3	4	4	5	1.00	0.041	1.00	Excellent
2–23 Deteriorating with other conditions, multiple conditions and/or complications that are not reversible; best available treatment has a poor outcome.	4	4	3	4	4	5	1.00	0.041	1.00	Excellent
3 − 1 Review current treatment and medication to make sure the person receives optimal care; minimise polypharmacy.	4	4	4	3	4	5	1.00	0.041	1.00	Excellent
3 − 2 Consider referral for specialist assessment if symptom or problems are complex and difficult to manage.	4	4	3	3	4	5	1.00	0.041	1.00	Excellent
3–3 Agree a current and future care plan with the person and their family/people close to them.	4	4	3	4	4	5	1.00	0.041	1.00	Excellent
3–4 Support carers.	4	4	3	3	4	5	1.00	0.041	1.00	Excellent
3–5 Plan ahead early if loss of decision-making capacity is likely.	4	4	3	3	4	5	1.00	0.041	1.00	Excellent
3–6 Record, share, and review care plans.	4	4	4	4	4	5	1.00	0.041	1.00	Excellent

*I-CVI*, Item-level Content Validity Index; *Pc*, chance agreement probability Evaluation criteria: fair = 0.40 to 0.59, good = 0.60 to 0.74, excellent > 0.74

#### Reliability

As shown in Table 3, the Cronbach’s Alpha was 0.76, indicating that the SPICT-CH exhibits acceptable internal consistency. When examining reliability among inter-rater agreement between nurses, the kappa value for each item (except for the kappa value of 0.22 for item 1-4) ranged from 0.41 to 0.63, indicating medium agreement among nurses on each item. However, the overall kappa value of 0.71 (95% CI 0.71-0.72, *P* = 0.000) demonstrated good agreement between assessing nurses.

**Table 3:** Rater agreement for the SPICT-CH

Item	Fleiss-kappa (95% Confidence interval)	*P*	Agreement	Disagreement
1–1	0.50 (0.49–0.50)	< 0.001	99.06%	0.94%
1–2	0.46 (0.45–0.46)	< 0.001	90.09%	9.91%
1–3	0.63 (0.63–0.64)	< 0.001	87.74%	12.26%
1–4	0.22 (0.22–0.23)	= 0.001	66.04%	33.96%
1–5	0.41 (0.41–0.42)	< 0.001	78.30%	21.70%
1–6	0.48 (0.47–0.48)	< 0.001	74.06%	25.94%
1–7	0.42 (0.42–0.43)	< 0.001	71.22%	28.78%
2 − 1	0.43 (0.43–0.44)	< 0.001	79.72%	20.28%
2–2	0.50 (0.50–0.51)	< 0.001	77.83%	22.17%
Overall	0.71 0.71–0.72	< 0.001	Positive 26.42% Negative 60.85%	12.74%

## Discussion

This is the first study to translate and validate a screening instrument in mainland China to support the early identification of individuals with a cancer diagnosis who would benefit from palliative care. In this study, we detailed the systematic methodological steps undertaken to translate the SPICT into Chinese and culturally adapt it, ensuring its relevance and validity in the Chinese context. An evaluation of the psychometric properties of SPICT-CH when used by clinicians among patients with cancer in a hospital setting showed that it has high levels of content validity, internal consistency and inter-rater reliability.

We undertook a rigorous and scientific process of translation and cross-cultural adaptation in modifying the SPICT for use in China, and we can be confident in its use to identify cancer patients who are suitable for palliative care in China. Since different religious beliefs and cultures will have an impact on the connotations expressed in the tool content, it is essential to address linguistic diversity and cultural differences to minimize their impact on outcome measurements [26]. During the translation and cross-cultural adaptation phase, we discussed clinically specialized vocabulary and terminology, particularly for indicators that lack defined criteria in the English version of the SPICT. Relevant criteria were added based on the healthcare context in mainland China, enabling a clearer description of the indicators and reducing cultural and linguistic differences in communication. Although some experts argued that certain indicators, such as “Depends on others for care due to increasing physical and/or mental health problems,” should add scores for self-care and psychological distress, we did not include any additional questions or items. This is because the SPICT-CH is an easy-to-use instrument that may support all types of clinicians in their daily work; adding too many questions would make it more complex and less efficient. Given the level of consistency among experts on the content during the adaptation phase of our study, we have confidence in the well-defined items of the SPICT-CH and its congruence with the construct it purports to measure. This result aligns with the findings reported in the Italian version of SPICT [27].

This study used two strategies of internal consistency and inter-rater reliability to assess the reliability of the SPICT-CH. Firstly, the instrument exhibited good internal consistency with a Cronbach’s Alpha of 0.76, indicating a good correlation between items. The internal consistency of SPICT-CH is slightly lower than that (Cronbach’s Alpha = 0.84) of the Chilean version of SPICT [28]. It may be due to differences in the age composition of the participants. Our study included a broad age range of the participants in our study, whereas the Chilean version of SPICT was exclusively tested on elderly patients. Our study found that SPICT-CH has an acceptable agreement between inter-rater reliability nurses, demonstrating a good stability of instrument. The inter-rater consistency (Kappa = 0.71) of SPICT-CH is higher than that (Kappa = 0.66) of the Thai version of SPICT [21]. It is possibly due to the different composition of assessors in the studies. In our study, assessors were exclusively nurses, while the validation study of the Thai version of SPICT included both doctors and nurses. Inter-rater reliability is a measure of the degree of agreement between different assessors in rating the same patients, and the different composition of assessors can impact the instrument’s reliability [25]. In addition, previous studies have indicated that doctors are more accurate than nurses in identifying individuals with palliative care needs. Therefore, differences in perspectives between doctors and nurses may be a contributing factor to the observed suboptimal inter-rater reliability. Overall, the SPICT-CH is a valuable instrument to identify patients in the inpatient setting and facilitate early initiation of palliative care.

In mainland China, a validated screening instrument capable of systematically identifying individuals with a cancer diagnosis requiring palliative care is of significant value. Such an instrument would enhance the accessibility to palliative care services and catalyze the advancement of palliative care infrastructure. The rise in the number of people who require palliative care in China due to changes in demographics and disease spectrum underscores the importance of this initiative [29]. While the Center to Advance Palliative Care (CAPC) recommends that palliative care should be provided in any settings based on patient needs, irrespective of the diagnosis and therapeutic goals [30], cancer patients constitute the largest group in need of palliative care in mainland China [31]. However, due to the influence of traditional Chinese culture and under-development of palliative care services as well as a medical specialty, most patients and their families lack sufficient knowledge about palliative care and have low motivation to engage in such care [32]. Furthermore, there are no clear referral criteria for palliative care to guide access in mainland China [33]. Therefore, to effectively meet the growing need for support for people suffering with advanced cancer, it is imperative to introduce resources and processes early to identify individuals with palliative care needs and a pathway for referral. The SPICT-CH has the potential as a screening instrument for early identifying individuals with palliative care needs. This could help to provide evidence-based support for establishing referral criteria for palliative care and improve the accessibility to palliative care in mainland China. Future research should focus on the accuracy in identifying patients with any advanced non-communicable diseases and how it can be embedded in routine clinical practice to improve palliative care coverage. In addition, current screening instruments are in the format of traditional paper-based. However, artificial intelligence applications such as ChatGPT and big data have developed rapidly, which can not only structuring data but also process unstructured data in the patient’s medical record system, and even images and pictures. These methods have the potential to significantly improve the efficiency and accuracy of identification, while reducing the burden on clinicians.

The feasibility of the instrument was also measured by its ease of use in routine clinical practice, including its acceptance, and feasibility of application [34]. In the pretest phase, we considered the amount of time needed to complete the assessment and the assessor’s comprehension of content. Our results indicated that the completion time of the instrument was acceptable, closely aligning with the time reported in previous studies [28, 35]. Additionally, assessors stated that the instrument was easy to understand and complete. Therefore, it is suitable to use SPICT-CH in mainland China’s busy routine clinical practices without imposing excessive workload on clinicians.

### Study strengths and limitations

Our major strength is that we used a rigorous cross-cultural approach in collaboration with multiple professionals from different disciplines to systematically adapt the SPICT for use in mainland China.

This study has some limitations. Firstly, we did not perform criterion validity due to the absence of a similar instrument serving as a gold standard to identify people needing palliative care in mainland China. Secondly, it is noted that the validation phase of SPICT-CH was exclusively conducted with nursing personnel. Consequently, we recommend caution when interpreting the validation results, given the limited scope of participant demographics. Although nurses are the healthcare professionals who have the most contact with patients, many nurses expressed that patients are more likely to communicate with their doctors about their treatment plans and needs. Therefore, future studies could recruit various healthcare professionals (e.g., nurses and physicians) to compare differences in their use of the SPICT-CH. Finally, the generalizability of the results is limited because we only recruited cancer patients in an inpatient setting for validation of SPICT-CH. Future studies could validate the effectiveness of SPICT-CH for patients with different diseases and from various healthcare settings.

## Conclusion

The Mandarin version of the SPICT has the potential to identify cancer patients with palliative care needs in a hospital setting. It has good content validity and acceptable internal consistency and inter-rater reliability. Therefore, the next step is to test the SPICT-CH in clinical daily practice, especially its accuracy in identifying the need for palliative care. Additionally, we recommend further studies to evaluate the effectiveness of SPICT-CH in diverse disease populations.

## Electronic supplementary material

Below is the link to the electronic supplementary material.

Supplementary Material 1

Supplementary Material 2

## Data Availability

Data is provided within the manuscript or supplementary information files.
