# Supplementary material for: Translation, cross-cultural adaptation and validation of the Chinese version of supportive and palliative care indicators tool (SPICT-CH) to identify cancer patients with palliative care needs
Source: BMC Palliat Care. 2025 Jan 7;24:4. doi: 10.1186/s12904-024-01641-x (PMC11708097; doi:10.1186/s12904-024-01641-x)
Supplement: Supplementary file 1 — Supplementary Material 1 [file 12904_2024_1641_MOESM1_ESM.docx]

Appendix S1The comparison of the original and Chinese version of the Supportive and Palliative Care Indicators Tool

|  | Original version | **Chinese version** | |
| --- | --- | --- | --- |
| **Name** | Supportive and Palliative Care Indicators Tool | 中文版支持性及安宁疗护服务需求指标工具 | |
| **instruction** | The SPICT is used to help identify people whose health is deteriorating.  Assess them for unmet supportive and palliative care needs.  Plan care | 安宁疗护是一种以患者需求及偏好为导向，确保患者能够获得个性化的症状管理和合适的照护以满足其需求和偏好的方法。它可以在疾病发展的早期阶段与治疗性的治疗手段同时进行以缓解痛苦等其他问题。如果能及时识别出需要安宁疗护的患者并为其及时提供安宁疗护，则可以为患者的临床结局带来更多积极的影响。本量表是用于早期识别有安宁疗护需求的非传染性疾病的患者，请您根据患者的病历和实际情况填写，每个条目的回答为“是”或“否”。 | |
| **1** | **Look for any general indicators of poor or deteriorating health.** | **健康状况不佳或恶化的通用指标** | |
| 1-1 | Unplanned hospital admission(s). | 非计划性反复入院 | □是 □否 |
| 1-2 | Performance status is poor or deteriorating, with limited reversibility. (e.g. The person stays in bed or in a chair for more than half the day.) | 活动状态差或正在恶化，逆转可能性低。（如，患者每天超过一半的时间卧床或做轮椅） | □是 □否 |
| 1-3 | Depends on others for care due to increasing physical and/or mental health problems. | 由于日益严重的身体问题，依赖他人照顾；  由于日益严重的心理健康或精神问题，需要心理或精神支持。 | □是 □否 |
|  |  |  | □是 □否 |
| 1-4 | The person’s carer needs more help and support. | 患者的照顾者需要比以往更多的帮助和支持。 | □是 □否 |

Appendix S1The comparison of the original and Chinese version of the Supportive and Palliative Care Indicators Tool (Continue)

|  | Original version | Chinese version |  |  |
| --- | --- | --- | --- | --- |
| 1-5 | Progressive weight loss; remains underweight; low muscle mass. | 体重持续下降；  维持低体重；  肌肉含量偏低。 | □是 □否 |  |
|  |  |  | □是 □否 |  |
|  |  |  | □是 □否 |  |
| 1-6 | Persistent symptoms despite optimal treatment of underlying condition(s). | 尽管对疾病进行了最佳治疗，但症状仍存在。 | □是 □否 |  |
| 1-7 | The person (or family) asks for palliative care; chooses to reduce, stop or not have treatment; or wishes to focus on quality of life. | 患者（或家属）表达安宁疗护的需求或意愿；  或患者（或家属）选择减少、停止或不进行治疗；  或患者（或家属）希望关注生活质量。 | □是 □否 |  |
|  |  |  | □是 □否 |  |
|  |  |  | □是 □否 |  |
| **2** | **Look for clinical indicators of one or multiple life-limiting conditions.** | **与特定疾病相关的终末期临床指标** | | |
|  | ***Cancer*** | ***癌症*** |  |  |
| 2-1 | Functional ability deteriorating due to progressive cancer. | 由于癌症进展，身体功能持续恶化。 | □是 □否 |  |
| 2-2 | Too frail for cancer treatment or treatment is for symptom control. | 身体太虚弱而无法接受癌症治疗；  治疗只是为了缓解症状。 | □是 □否 |  |
|  |  |  | □是 □否 |  |
|  | ***Dementia/frailty*** | ***痴呆症/虚弱*** |  |  |
| 2-3 | Unable to dress, walk or eat without help. | 在没有帮助的情况下无法穿衣、行走或进食。 | □是 □否 |  |

Appendix S1The comparison of the original and Chinese version of the Supportive and Palliative Care Indicators Tool (Continue)

|  | Original version | Chinese version |  |
| --- | --- | --- | --- |
| 2-4 | Eating and drinking less; difficulty with swallowing. | 进食量和饮水量减少；  或吞咽困难。 | □是 □否 |
|  |  |  | □是 □否 |
| 2-5 | Urinary and faecal incontinence. | 大小便失禁。 | □是 □否 |
| 2-6 | Not able to communicate by speaking; little social interaction. | 不能用言语交流；  或几乎没有社交。 | □是 □否 |
|  |  |  | □是 □否 |
| 2-7 | Frequent falls; fractured femur. | 频繁跌倒；  或因跌倒导致股骨骨折。 | □是 □否 |
|  |  |  | □是 □否 |
| 2-8 | Recurrent febrile episodes or infections; aspiration pneumonia. | 反复性发热；  或反复性感染；  或吸入性肺炎。 | □是 □否 |
|  |  |  | □是 □否 |
|  |  |  | □是 □否 |
|  | ***Neurological disease*** | ***神经系统疾病*** |  |
| 2-9 | Progressive deterioration in physical and/or cognitive function despite optimal therapy. | 尽管进行了最佳治疗，但身体和/或认知功能仍在持续恶化。 | □是 □否 |
| 2-10 | Speech problems with increasing difficulty communicating and/or progressive difficulty with swallowing. | 语言沟通困难和/或吞咽困难持续加重 | □是 □否 |

Appendix S1The comparison of the original and Chinese version of the Supportive and Palliative Care Indicators Tool (Continue)

|  | Original version | Chinese version |  |
| --- | --- | --- | --- |
| 2-11 | Recurrent aspiration pneumonia; breathless of respiratory failure. | 反复性吸入性肺炎；  呼吸困难或呼吸衰竭。 | □是 □否 |
|  |  |  | □是 □否 |
| 2-12 | Persistent paralysis after stroke with significant loss of function and ongoing disability. | 卒中后持续瘫痪，并伴有严重的失能。 | □是 □否 |
|  | ***Heart/vascular disease*** | ***心脏/血管疾病*** |  |
| 2-13 | Heart failure or extensive, untreatable coronary artery disease; with breathless or chest pain at rest or on minimal effort. | 尽管进行了最佳的治疗仍然不能改善的心力衰竭或广泛的、无法治疗的冠状动脉疾病；  或在休息时或在最小活动量时有呼吸困难或胸痛。 | □是 □否 |
|  |  |  | □是 □否 |
| 2-14 | Severe, inoperable peripheral vascular disease. | 严重的、无法手术的外周血管疾病。 | □是 □否 |
|  | ***Respiratory disease*** | ***呼吸系统疾病*** |  |
| 2-15 | Severe, chronic lung disease; with breathlessness at rest or on minimal effort between exacerbations. | 严重的慢性肺部疾病；  或在休息时或在两次病情加重的间隙进行最小活动量的活动时，有呼吸困难的症状。 | □是 □否 |
|  |  |  | □是 □否 |
| 2-16 | Persistent hypoxia needing long term oxygen therapy. | 持续缺氧（氧分压低于90mmhg），需要长期氧气治疗。 | □是 □否 |

Appendix S1The comparison of the original and Chinese version of the Supportive and Palliative Care Indicators Tool (Continue)

|  | Original version | Chinese version |  |
| --- | --- | --- | --- |
| 2-17 | Has needed ventilation for respiratory failure or ventilation is contraindicated. | 曾因呼吸衰竭需要通气，或通气有禁忌症。 | □是 □否 |
|  | ***Kidney disease*** | ***肾脏疾病*** |  |
| 2-18 | Stage 4 or 5 chronic kidney disease (eGFR<30ml/min) with deteriorating health. | 伴随健康状况恶化的4或5期慢性肾脏病（eGFR<30ml/min）。 | □是 □否 |
| 2-19 | Kidney failure complicating other life limiting conditions or treatments. | 肾脏衰竭并发其他生命限制性疾病或治疗。 | □是 □否 |
| 2-20 | Stopping or not starting dialysis. | 停止或无法透析。 | □是 □否 |
|  | ***Liver disease*** | ***肝脏疾病*** |  |
| 2-21 | Cirrhosis with one or more complications in the past year:   - diuretic resistant ascites - hepatic encephalopathy - hepatorenal syndrome - bacterial peritonitis - recurrent variceal bleeds | 过去一年中有肝硬化，并伴有以下一个或多个并发症。   - 利尿剂抵抗性腹水 - 肝性脑病 - 肝肾综合征 - 细菌性腹膜炎 - 反复性静脉曲张出血 | □是 □否 |
| 2-22 | Liver transplant is not possible. | 无法或不适合进行肝脏移植；  或肝脏移植失败。 | □是 □否 |
|  |  |  | □是 □否 |

Appendix S1The comparison of the original and Chinese version of the Supportive and Palliative Care Indicators Tool (Continue)

|  | ***Other conditions*** | ***其他*** |  |
| --- | --- | --- | --- |
| 2-23 | Deteriorating with other conditions, multiple conditions and/or complications that are not reversible; best available treatment has a poor outcome. | 伴有其他，多种和/或并发症出现不可逆转的恶化；  或已获得最佳治疗但效果仍然不佳。 | □是 □否 |
|  |  |  | □是 □否 |
| NA | | **注：当患者符合2个健康状况不佳或恶化的通用指标＋1个与特定疾病相关的终末期临床指标时，则认为SPICT-CH阳性，可考虑为安宁疗护服务适用人群，此时请完成第3部分的内容。** | |
| **3** | **Review current care and care planning.** | **回顾目前的医疗照护方案，讨论并制订未来医疗照护计划** | |
| 3-1 | Review current treatment and medication to make sure the person receives optimal care; minimise polypharmacy. | 审查目前的治疗和用药，以确保患者得到最佳的护理；  或尽量减少多种药物联合使用。 | □是 □否 |
|  |  |  | □是 □否 |
| 3-2 | Consider referral for specialist assessment if symptom or problems are complex and difficult to manage. | 如果症状或问题较复杂且较难管理，可以考虑转诊至多学科团队评估。 | □是 □否 |
| 3-3 | Agree a current and future care plan with the person and their family/people close to them. | 与患者及其家属协商当前的治疗方案和未来的照护计划（预立医疗照护计划）。 | □是 □否 |
| 3-4 | Support carers. | 支持照顾者。 | □是 □否 |
| 3-5 | Plan ahead early if loss of decision-making capacity is likely. | 如果患者有可能丧失决策能力，应及早做好相应计划。 | □是 □否 |
| 3-6 | Record, share, and review care plans. | 记录，分享和回顾照护计划。 | □是 □否 |

Appendix S2 demographic characteristics about the expert panel

| Number | Educational status | Age (years) | Position | Research field | Time of Working in this field (years) |
| --- | --- | --- | --- | --- | --- |
| A | Doctor | 30 | Assistant Professor | Cancer Prevention and Screening | 5 |
| B | Doctor | 40 | Lecturer | Palliative care | 9 |
| C | Master | 36 | Attending Physician | Palliative care | 10 |
| D | Bachelor | 60 | Medical Director | Palliative care | 18 |
| E | Doctor | 37 | Co-Chief Superintendent Nurse | Palliative care | 15 |
